# Supplementary material for: Bell’s theorem for temporal order
Source: Nat Commun. 2019 Aug 21;10:3772. doi: 10.1038/s41467-019-11579-x (PMC6704104; doi:10.1038/s41467-019-11579-x)
Supplement: Supplementary file 2 — Supplementary Information [file 41467_2019_11579_MOESM2_ESM.pdf]

Suplementary Information

Bell's theorem for temporal order

Zych et al.

Supplementary Note 1: Causally non-separable quantum processes

Non-classical causal relations can be studied within a recent framework for quantum mechanics with no pre-defined causal structure introduced in ref. [1]. The starting point of the framework is the notion of local events that take place in local regions, with spatial and temporal boundaries of the region defined by local clocks. An event is identified with an operation performed in the local region (for example a unitary transformation, or a projection on a given state obtained as the result of a measurement). A physical scenario, comprising the space-time geometry in which the local regions are embedded, the initial state, and the dynamics connecting the regions, is compactly represented by a process—a specification of the probabilities for any possible event/local operation to take place in each region.

At a formal level, a local region X is defined by an input Hilbert space $\mathcal{H}^{\mathrm{X_I}}$ and an output Hilbert space $\mathcal{H}^{\mathrm{X_O}}$, identified with the quantum degrees of freedom on space-like surfaces on the past and future of X, respectively. Quantum operations are represented as operators $M^{\mathrm{X_I X_O}} \in \mathcal{L}(\mathcal{H}^{\mathrm{X_I}}) \otimes \mathcal{L}(\mathcal{H}^{\mathrm{X_O}})$, where $\mathcal{L}(\mathcal{H})$ is the space of linear operators on the Hilbert space $\mathcal{H}$. Probabilities for events in regions A, B,... are then given by a generalisation of the Born rule:

$$P(M^{\mathrm{A_I A_O}}, M^{\mathrm{B_I B_O}}, \dots) = \mathrm{Tr}\left[\left(M^{\mathrm{A_I A_O}} \otimes M^{\mathrm{B_I B_O}} \otimes \dots\right) \cdot W^{\mathrm{A_I A_O B_I B_O} \cdots}\right], \tag{1}$$

where $W^{\mathrm{A_I A_O B_I B_O} \cdots} \in \mathcal{L}(\mathcal{H}^{\mathrm{A_I}}) \otimes \mathcal{L}(\mathcal{H}^{\mathrm{A_O}}) \otimes \mathcal{L}(\mathcal{H}^{\mathrm{B_I}}) \otimes \mathcal{L}(\mathcal{H}^{\mathrm{B_O}})$ is the process matrix.

In this formalism, causal relations between local regions are encoded in the process matrix. For example, the process matrix

$$W^{\mathrm{A_I A_O B_I B_O}} = \rho^{\mathrm{A_I}} \otimes [[\mathbb{1}]]^{\mathrm{A_O B_I}} \otimes \mathbb{1}^{\mathrm{B_O}}, \text{ where} \tag{2}$$

$$[[\mathbb{1}]]^{\mathrm{A_O B_I}} := |\mathbb{1}\rangle\rangle\langle\langle\mathbb{1}|^{\mathrm{A_O B_I}} \text{ and} \tag{3}$$

$$|\mathbb{1}\rangle\rangle^{\mathrm{A_O B_I}} := \sum_j |j\rangle^{\mathrm{A_O}} |j\rangle^{\mathrm{B_I}}, \tag{4}$$

represents a situation where an agent at A receives a state ρ, while the output of A's operation is sent to B through the identity channel. Such a process is only compatible with the order of events A $\preceq$ B; more general processes compatible with an order of events given by a permutation σ are denoted W^{σ}. If the order is determined by a classical variable λ, defined in some region in the past of all events, the process matrix has the form

$$W = \int d\lambda W^{\sigma_\lambda} P(\lambda) \tag{5}$$

for some probability distribution $P(\lambda)$.

The question of whether a certain quantum scenario can be embedded in a classical space-time, with a classical order of events, thus reduces to the question whether the corresponding process matrix can be decomposed in a mixture of the form (5), which we call causally separable. (A more general

definition, where the order of future events can depend on past events, is not necessary for our analysis.) The quantum switch, described in the main text, section “Quantum control of temporal order”, is represented by the process matrix

$$W = |\omega\rangle\langle\omega| \tag{6}$$

$$|\omega\rangle = \frac{1}{\sqrt{2}}\left(|\mathrm{K}_{\mathrm{A}\prec\mathrm{B}}\rangle^{\mathrm{M_I}}|\mathrm{ABC}\rangle + |\mathrm{K}_{\mathrm{B}\prec\mathrm{A}}\rangle^{\mathrm{M_I}}|\mathrm{BAC}\rangle\right), \tag{7}$$

$$|\mathrm{ABC}\rangle = |\psi\rangle^{\mathrm{A_I}}|\mathbb{1}\rangle\!\rangle^{\mathrm{A_O B_I}}|\mathbb{1}\rangle\!\rangle^{\mathrm{B_O C_I}}, \quad |\mathrm{BAC}\rangle = |\psi\rangle^{\mathrm{B_I}}|\mathbb{1}\rangle\!\rangle^{\mathrm{B_O A_I}}|\mathbb{1}\rangle\!\rangle^{\mathrm{A_O C_I}}, \tag{8}$$

where M labels the control system and C is the region where the system is measured after the operations in regions A, B are performed. As shown in ref. [2], it is possible to find an experimental procedure, namely a set of operations and measurements for A, B, C, M, that allows proving the causal non-separability of the switch. However, such a causal witness is both device and theory dependent, namely it relies on the quantum description of the operations performed. Causal inequalities [1, 3], on the other hand, provide a device and theory independent test for causal order; however, no quantum-control of causal order can violate causal inequalities, as proven in refs. [2, 4], and it is an open question whether any physically realisable process can.

The process matrix corresponding to the scenario with entangled temporal orders, introduced in the main text is

$$W = |\varpi\rangle\langle\varpi| \tag{9}$$

$$|\varpi\rangle = \frac{1}{\sqrt{2}}\left(|\mathrm{K}_{\mathrm{A}\prec\mathrm{B}}\rangle^{\mathrm{M_I}}|\mathrm{A_1B_1C_1}\rangle|\mathrm{A_2B_2C_2}\rangle + |\mathrm{K}_{\mathrm{B}\prec\mathrm{A}}\rangle^{\mathrm{M_I}}|\mathrm{B_1A_1C_1}\rangle|\mathrm{B_2A_2C_2}\rangle\right), \tag{10}$$

using definitions similar to (8). Just as for the switch, it is easy to prove that process (9) is not causally separable: As it is a rank-one projector, it cannot be decomposed as a non-trivial mixture of orders; Yet it does not describe a process with a definite order, because the signalling relations between parties do not define a partial order. However, a process of this type cannot be used to violate causal inequalities, see e.g. ref. [2]. The procedure described in the main text can nonetheless prove the causal non-separability of process (9) in a theory-independent, albeit device-dependent, way.

Supplementary Note 2: State and measurements for the CHSH inequality violation

Consider a two-qubit system in an initial state $|\psi_1\rangle^{\mathrm{S_1}} \otimes |\psi_2\rangle^{\mathrm{S_2}} \equiv |\mathrm{z}+\rangle^{\mathrm{S_1}} \otimes |\mathrm{z}+\rangle^{\mathrm{S_2}}$ and unitaries

$$U_{\mathrm{A_1}} = U_{\mathrm{A_2}} \equiv U_{\mathrm{A}} = \frac{\mathbb{1} + i\sigma_{\mathrm{x}}}{\sqrt{2}}, \qquad U_{\mathrm{B_1}} = U_{\mathrm{B_2}} \equiv U_{\mathrm{B}} = \sigma_{\mathrm{z}}, \tag{11}$$

with σ_{x} and σ_{z} the Pauli matrices. We find $U_{\mathrm{A}}U_{\mathrm{B}} = \frac{\sigma_{\mathrm{z}}+\sigma_{\mathrm{y}}}{\sqrt{2}}$ and $U_{\mathrm{B}}U_{\mathrm{A}} = \frac{\sigma_{\mathrm{z}}-\sigma_{\mathrm{y}}}{\sqrt{2}}$, and the final state (eq. (8) in the main text) reads

$$\frac{1}{\sqrt{2}}\left(|\mathrm{x}+\rangle^{\mathrm{S_1}}|\mathrm{x}+\rangle^{\mathrm{S_2}} \pm |\mathrm{x}-\rangle^{\mathrm{S_1}}|\mathrm{x}-\rangle^{\mathrm{S_2}}\right). \tag{12}$$

Importantly, the sign above depends on the result $|\pm\rangle$ of the measurement on the massive system. In order to violate Bell inequalities, agent c_1 measures $\mathcal{C}_1^0 = \frac{\sigma_y - \sigma_z}{\sqrt{2}}$ for setting $i_1 = 0$ and observable $\mathcal{C}_1^1 = \frac{\sigma_y + \sigma_z}{\sqrt{2}}$ for $i_1 = 1$, while agent c_2 measures observable $\mathcal{C}_2^0 = \sigma_y$ for setting $i_2 = 0$ and $\mathcal{C}_2^1 = \sigma_z$ for $i_2 = 1$. The expectation value of the CHSH correlation [5] with the above measurement choices is

$$\langle \text{CHSH} \rangle_\pm = \left\langle \mathcal{C}_1^0 \otimes \mathcal{C}_2^0 + \mathcal{C}_1^0 \otimes \mathcal{C}_2^1 + \mathcal{C}_1^1 \otimes \mathcal{C}_2^0 - \mathcal{C}_1^1 \otimes \mathcal{C}_2^1 \right\rangle_\pm = \mp 2\sqrt{2}, \tag{13}$$

for the two outcomes $z = \pm 1$ of the measurement at D. This means that conditioned on the value of z, the measurements at C_1 and C_2 violate the CHSH inequality $|\langle \text{CHSH} \rangle| \leq 2$. Importantly, the settings at C_1 and C_2 are independent of z and therefore the three measurements can be performed at space-like separation. The violation of CHSH inequality can be verified once all the data are compared.

Supplementary Note 3: Realisations of the protocol

To achieve large time dilation between a pair of clocks one can use a heavy object or, alternatively, any mass M dense enough to put one of the clocks close to its Schwarzschild radius $R_{\text{Sch}} := \frac{2GM}{c^2}$. The ticking rate of a clock at $R_{\text{Sch}} + \epsilon$ with $\epsilon \ll R_{\text{Sch}}$ differs from the ticking rate of an identical clock at $R_{\text{Sch}} + l$, $l > \epsilon$, by a factor approximately $\sqrt{\frac{R_{\text{Sch}}}{\epsilon}\left(1 + 2\frac{\Phi(R_{\text{Sch}}+l)}{c^2}\right)}$, which becomes arbitrarily large for a small ϵ. Practical realisation of the protocol in Figure 3 in the main text will nevertheless pose a formidable challenge, but it is in principle possible. Below we give an example.

Consider configurations $K_{A \prec B}$, $K_{B \prec A}$ realised using an effectively point-like body with a fixed mass. The distance between agents b_i, $i = 1, 2$ and the mass is the same for both $K_{A \prec B}$ and $K_{B \prec A}$, while agents a_i are closer to the mass in configuration $K_{B \prec A}$ than in $K_{A \prec B}$, see Fig. 1 a). The subsystems S_i can be realised as two identically prepared, uncorrelated photons and the local operations can be performed on their polarisation degrees of freedom (DOF). The photon source is equally distant from $K_{A \prec B}$ and $K_{B \prec A}$, (equidistant to the extent that the local clock of the source remains sufficiently uncorrelated with the mass). All clocks involved in the protocol are initially synchronised with the clock of the source.

At a pre-defined time T_s according to the clock at the source, the source emits the photon pair – the emission time is thus uncorrelated with the mass configuration. Photon S_i is directed towards agent a_i, then to b_i, again to a_i, back to b_i, and exits towards agent c_i (or c_i simply replaces a_i), see Fig. 1 b). The agents interact with the relevant DOF of the photon only once, at the time τ^* as measured by their local clocks. The unitary transformations are assumed to be independent of the mass configuration (or other aspects of the experiment). As discussed in the main text, the emission time T_s of the photons can be chosen such that event A_i (at which U_{A_i} is applied) is before the event B_i (at which U_{B_i} is applied) and so that event A_i coincides with the photon reaching a_i for the

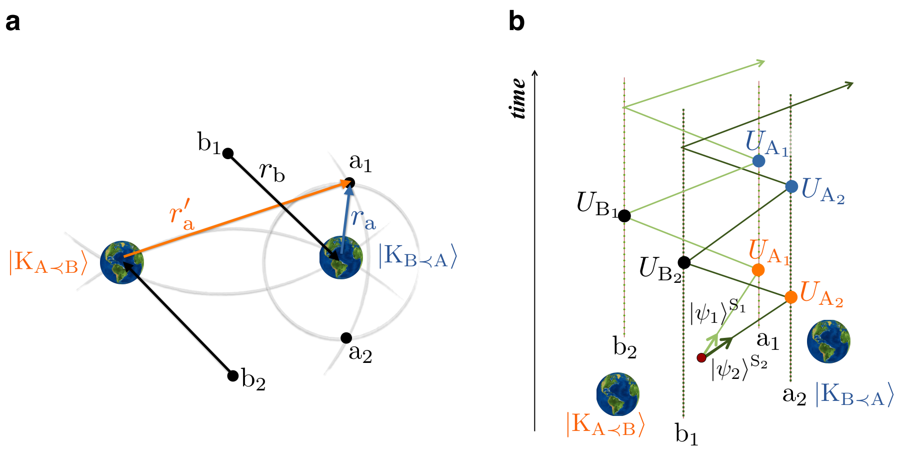

Supplementary Figure 1: Protocol for a violation of Bell's inequalities for temporal order using polarisation states of photons. **a** Mass configurations $K_{A\prec B}$, $K_{B\prec A}$ and location of the agents $a_i, b_i, i = 1, 2$. b_i are at a distance r_b from both configurations, while a_i are at a distance r_a from $K_{B\prec A}$ and $r'_a > r_a$ from $K_{A\prec B}$. **b** Space-time diagram of the protocol. Systems S_1, S_2 are implemented in the polarisation of two photons, initially in a product state $|\psi_1\rangle^{S_1}|\psi_2\rangle^{S_2}$. Green lines are the photons' world lines; green dotted lines are world lines of the agents. Orange (blue) dots mark events when agents a_i apply unitaries U_{A_i} for the configuration $K_{A\prec B}$ ($K_{B\prec A}$); black dots mark events when b_i apply U_{B_i}. The photons bounce twice between the agents, but each operation is applied on the photon only once – when the local clocks of the agents show proper time τ^*. Due to time dilation induced by the mass, U_{A_i} are applied before U_{B_i} for configuration $K_{A\prec B}$ (and the events A_i coincide with the photons reaching a_i for the first time) – and are applied after U_{B_i} for configuration $K_{B\prec A}$ (and the events A_i coincide with the photons reaching a_i for the second time). Reprinted by permission from Springer Customer Service Centre GmbH: Springer International Publishing "Quantum Systems under Gravitational Time Dilation" by M. Zych (2017).

first time for configuration $K_{A\prec B}$, and when the photon reaches a_i for the second time for $K_{B\prec A}$. The photon reaches a_i twice, before or after τ^* – depending on the mass configuration, at which no operation is performed: The photon is reflected with no transformation on the polarisation. The event when the operation U_{B_i} is applied always coincides with the photon reaching b_i for the first time, since agents b_i are at the same distances to the mass for both configurations.

In general, the travel time of the photon can depend on the mass configuration due to the Shapiro delay [6, 7]. In order to mitigate this effect, after the emission time T_s – sufficient to induce the required time dilation between the clocks – the mass can be (coherently) moved such that it is at the same distance from each agent (for both $K_{A\prec B}$ and $K_{B\prec A}$), or such that it is sufficiently far away from both. Moreover, in order to de-correlate the time-dilated clocks from the systems S_i, the amplitudes of the mass can be swapped and the mass can be measured by the agent d after a time interval equal to T_s – when the clocks of a_i and b_i become synchronised again. (We note that Methods section provides details of a protocol that provides both: suppression of the Shapiro effect and decorrelation of the clocks).

Here we discuss another possibility for the realisation of the protocol. The mass distribution is

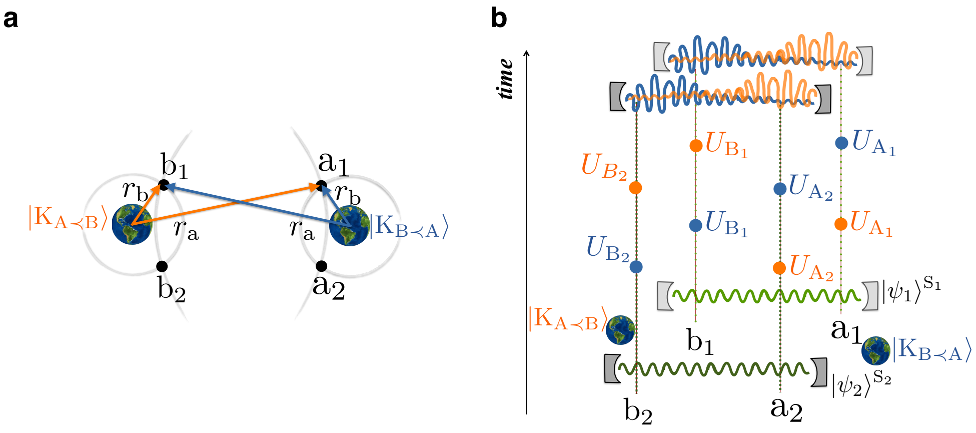

Supplementary Figure 2: Protocol for the violation of Bell's inequalities for temporal order with spatial modes of a quantum field. **a** Mass configurations: In configuration $\mathrm{K}_{\mathrm{A}\prec\mathrm{B}}$ the mass is at the same distance r_b from both b_i and at a distance $r_\mathrm{a} > r_\mathrm{b}$ from both a_i. For the configuration $\mathrm{K}_{\mathrm{B}\prec\mathrm{A}}$ the mass is at r_a from both b_i, and at r_b from both a_i. **b** Space-time diagram. Systems S_i are implemented in two regions of an electromagnetic field, prepared in a vacuum state. Agents apply coarse-grained operations $U_{\mathrm{A}_i}, U_{\mathrm{B}_i}$ (in the subspace spanned by the vacuum and a single-photon state) locally on the field at correspondingly marked events. For configuration $\mathrm{K}_{\mathrm{A}\prec\mathrm{B}}$ ($\mathrm{K}_{\mathrm{B}\prec\mathrm{A}}$) only orange (blue) events occur, and the final state of the field is represented in orange (blue). Reprinted by permission from Springer Customer Service Centre GmbH: Springer International Publishing "Quantum Systems under Gravitational Time Dilation" by M. Zych (2017).

such that for $\mathrm{K}_{\mathrm{A}\prec\mathrm{B}}$ the mass is closer to b_1 than to a_1 and for $\mathrm{K}_{\mathrm{B}\prec\mathrm{A}}$ the relative distances are reversed. The same holds for agents $\mathrm{a}_2, \mathrm{b}_2$ who are placed symmetrically to agents $\mathrm{a}_1, \mathrm{b}_1$ with respect to the mass; see Supplementary Figure 2 a).

The local operations can be performed in the Fock space of a photon field, more precisely in the two-level subspace spanned by the vacuum and a single-photon state of a chosen field mode. The field is prepared in some mode α at event A_1, and in mode β at event B_1. The modes are chosen such, that the two final states of the field at event C_1 – obtained depending on the order between events $\mathrm{A}_1, \mathrm{B}_1$ – are distinguishable. The situation for the agents $\mathrm{a}_2, \mathrm{b}_2$ is the same and they prepare the modes α, β, at the events $\mathrm{A}_2, \mathrm{B}_2$, respectively, see Supplementary Figure 2 b).

One needs to note, that the vacuum state of a relativistic quantum field is entangled with respect to the local subsystems [8, 9, 10]. However, this entanglement is effectively inaccessible under coarse-grained operations [11]. Thus, if the operations performed by the agents are sufficiently coarse-grained, the initial state consisting of the local regions of a vacuum of a quantum field is effectively separable and does not violate the assumptions of the protocol. This implementation differs from the example given in the main text in that it does not need a source that would produce a state at a specific time, and distribute it to the agents.

Supplementary Note 4: Gravitation vs other methods for a quantum control of temporal order

The superposition or entanglement of temporal orders was discussed here in the context where a far away agent prepares a quantum state of a massive system which due to relativistic gravity effects yields a desired quantum causal structure for future events. There are, however, other methods to control the temporal order between operations applied on a system. One possibility is to control the positions of clocks that define when the operations are applied. For example, by placing clock a closer and b further away from a fixed mass in superposition with b closer and a further, proper times of the clocks become entangled as in the gravitational switch. Quantum control of the time order can also be achieved without any use of gravitational interaction, e.g. in the extended model of a quantum circuit: Quantum gates can be applied on a system in different orders in a superposition [12, 13, 14, 15] The latter has already been practically implemented using an interferometer to route a photon through two gates (acting on its polarisation) in different orders [16, 17, 18, 19, 20, 21, 22]

The key difference between the gravitational scenario presented in this work and other schemes is that, in the latter, the events would be embedded in a classical space-time: In the example of an entangled clock-pair, only these specific clocks could be used to label events for which temporal order is non-classical, while any other nearby clock would define classically ordered events. In the example of an extended quantum circuit, only the photon that went through a beam splitter will undergo different transformations in a non-classical order. In contrast, in the scenario considered in this work *any* local system in the spacetime region affected by the superposition state of the mass will have classically undefined proper time. Thus, any two pairs of clocks in this region will define events with an entangled order.

The above can be highlighted by considering the scenario leading to the violation of the Bell-like inequality for temporal order in a quantum coordinate system [23] defined relative to the massive body, which is here in superposition. (A complete theory of such quantum coordinate transformations is missing; however, they were also studied within the approach of quantum reference frames in ref. [24], where non-relativistic transformations between quantum reference bodies in relative superposition states were constructed such that the dynamics of the systems of interest is described relative to other physical systems, rather than relative to an idealised notion of coordinates.) Space-time coordinates of the events would then be defined with respect to the position of the mass and a local clock at its location – rather than with respect to the positions and proper times of the clocks of the agents. By definition, in these coordinates the location of the mass would be fixed. As a result, all local operations performed in the local regions of the agents would appear to be embedded in a fixed space-time metric but performed at different space-time events in superposition – such that the orders of events in different space-time regions are always entangled.

Another manifestation of the classical space-time underlying other implementations is that local

measurements can reveal the order of events. For the two examples discussed above, this could be achieved, respectively, by non-demolition measurements of the positions of the clocks or the photon's time of arrival to a gate in a circuit. On the contrary, in the gravitational scenario introduced in this work, all operations are performed at fixed local times, independently of the event order. No local time measurement can reveal whether a given agent is acting first or second. It is further possible to consider a realisation of our scheme where no local measurement, temporal or otherwise, can reveal which of the two mass configurations was prepared, and thus what the event order was. This can be achieved by using mass distributions that do not exert any force on the laboratories, but still cause the necessary time dilation. For example, each laboratory can be placed inside a spherical mass shell [25]. The gravitational potential inside the shell is constant and depends on the mass and radius of the shell. Therefore, laboratories inside shells of different radii experience relative time dilation but no force if the two shells are sufficiently far from each other. All steps of the protocol described in the main text can be reproduced using spherical shells: configurations leading to different temporal order are obtained by placing a shell with smaller radius either around laboratory a or b. The swap of the mass positions would then be replaced by exchange of the shell's radii. This realisation has the further advantage that the laboratories are in free fall and no mass-configuration-dependent acceleration is needed to keep them on the chosen world lines. Note that, even if the gravitational force outside a shells is taken into account, and thus the laboratories might need to accelerate to maintain the desired trajectories, these accelerations do not need to depend on the mass configuration. This is because outside a shell of a fixed mass the same force is exerted at a given distance from its centre-of-mass, independently of the shell's radius. The laboratories would experience the same gravitational acceleration for each mass configuration, and thus their world lines can still be defined independently of the configuration of mass. For interferometry with massive shells see refs [26, 27].

The above discussion shows that there is a fundamental difference between the gravitational control of temporal order discussed here and other methods. Although the final state of a system undergoing some transformations in a non-classical order is independent of how the order was controlled, only when the mass controls temporal relations is the effect universal – applying to all events in some space-time region. Thus, only in the gravitational case one would conclude that non-classical temporal order indicates non-classicality of space-time.

Supplementary Note 5: What if it is fundamentally not possible to violate the Bell inequality for time order?

Since a test of Bell's inequalities for temporal order has never been performed and would be very challenging, one can also ask what if it is not possible even in principle to violate the corresponding Bell's inequalities, or if it is fundamentally not possible to satisfy assumptions of the theorem other

than 3? If that were the case, a classical description of temporal order could always be given, e.g. in terms of the classical variable λ (introduced in Definition 1 in the main text) – even in space-times originating from a quantum state of a massive body. Moreover, the classical variable describing temporal order of events could be used to define a classical time parameter according to which the systems evolve, even in scenarios involving macroscopic masses in quantum superposition states. Interestingly, this would imply that models forbidding spatial superpositions of large masses on the ground that it is not possible to define time evolution in the resulting space-time, such as refs. [28, 29, 30, 31, 32] are redundant: Time would be compatible with a classical description (in terms of a hidden variable) even in the presence of quantum states of massive bodies.

Supplementary References

[1] Oreshkov, O., Costa, F. M. & Brukner, C. Quantum correlations with no causal order. *Nature Communications* **3**, 1092 (2012).

[2] Araújo, M. *et al.* Witnessing causal nonseparability. *New Journal of Physics* **17**, 102001 (2015).

[3] Branciard, C., Araújo, M., Feix, A., Costa, F. & Brukner, Č. The simplest causal inequalities and their violation. *New. J. Phys.* **18**, 013008 (2016).

[4] Oreshkov, O. & Giarmatzi, C. Causal and causally separable processes. *New Journal of Physics* **18**, 093020 (2016).

[5] Clauser, J. F., Horne, M. A., Shimony, A. & Holt, R. A. Proposed experiment to test local hidden-variable theories. *Physical Review Letters* **23**, 880–884 (1969).

[6] Shapiro, I. I. Fourth test of general relativity. *Physical Review Letters* **13**, 789–791 (1964).

[7] Shapiro, I. I. *et al.* Fourth test of general relativity: New radar result. *Physical Review Letters* **26**, 1132–1135 (1971).

[8] Reeh, H. & Schlieder, S. Bemerkungen zur unitäräquivalenz von lorentzinvarianten feldern. *Il Nuovo Cimento* **22**, 1051–1068 (1961).

[9] Summers, S. J. & Werner, R. Bell's inequalities and quantum field theory. I. General setting. *Journal of Mathematical Physics* **28**, 2440–2447 (1987).

[10] Summers, S. J. & Werner, R. Bell's inequalities and quantum field theory. II. Bell's inequalities are maximally violated in the vacuum. *Journal of Mathematical Physics* **28**, 2448–2456 (1987).

[11] Zych, M., Costa, F., Kofler, J. J. & Brukner, C. Entanglement between smeared field operators in the Klein-Gordon vacuum. *Physical Review D* **81**, 125019 (2010).

[12] Chiribella, G., D'Ariano, G. M., Perinotti, P. & Valiron, B. Quantum computations without definite causal structure. *Physical Review A* **88**, 022318 (2013).

[13] Chiribella, G. Perfect discrimination of no-signalling channels via quantum superposition of causal structures. *Physical Review A* **86**, 040301 (2012).

[14] Colnaghi, T., D'Ariano, G. M., Facchini, S. & Perinotti, P. Quantum computation with programmable connections between gates. *Physics Letters A* **376**, 2940–2943 (2012).

[15] Araújo, M., Costa, F. & Brukner, C. Computational advantage from quantum-controlled ordering of gates. *Physical Review Letters* **113**, 250402 (2014).

[16] Procopio, L. M. *et al.* Experimental superposition of orders of quantum gates. *Nature Communications* **6**, 7913 (2015).

[17] Rubino, G. *et al.* Experimental verification of an indefinite causal order. *Science Advances* **3** (2017).

[18] Rubino, G. *et al.* Experimental entanglement of temporal orders. *Preprint at http://arxiv.org/abs/1712.06884* (2017).

[19] Goswami, K. *et al.* Indefinite causal order in a quantum switch. *Physical Review Letters* **121**, 090503 (2018).

[20] Goswami, K., Romero, J. & White, A. Communicating via ignorance. *Preprint at https://arxiv.org/abs/1807.07383* (2018).

[21] Wei, K. *et al.* Experimental quantum switching for exponentially superior quantum communication complexity. *Physical Review Letters* **122**, 120504 (2019).

[22] Guo, Y. *et al.* Experimental investigating communication in a superposition of causal orders. *Preprint at http://arxiv.org/abs/1811.07526* (2018).

[23] Zych, M., Costa, F. & Ralph, T. C. Relativity of quantum superpositions. *Preprint at https://arxiv.org/abs/1809.04999* (2018).

[24] Giacomini, F., Castro-Ruiz, E. & Brukner, Č. Quantum mechanics and the covariance of physical laws in quantum reference frames. *Nature Communications* **10**, 494 (2019).

[25] Hohensee, M. A., Estey, B., Hamilton, P., Zeilinger, A. & Müller, H. Force-Free Gravitational Redshift: Proposed Gravitational Aharonov-Bohm Experiment. *Physical Review Letters* **108**, 230404 (2012).

[26] Gooding, C. & Unruh, W. G. Bootstrapping time dilation decoherence. *Foundations of Physics* 1–13 (2015).

[27] Gooding, C. & Unruh, W. G. Self-gravitating interferometry and intrinsic decoherence. *Physical Review D* **90**, 044071 (2014).

[28] Karolyhazy, F. Gravitation and quantum mechanics of macroscopic objects. *Il Nuovo Cimento A* **42**, 390–402 (1966).

[29] Diosi, L. Models for universal reduction of macroscopic quantum fluctuations. *Physical Review A* **40**, 1165–1174 (1989).

[30] Penrose, R. On gravity's role in quantum state reduction. *General Relativity and Gravitation* **28**, 581–600 (1996).

[31] Stamp, P. C. E. Environmental decoherence versus intrinsic decoherence. *Phil. Trans. R. Soc. A* **370**, 4429–4453 (2012).

[32] Penrose, R. On the gravitization of quantum mechanics 1: Quantum state reduction. *Foundations of Physics* **44**, 557–575 (2014).
